# Assessing parental comprehension of online resources on childhood pain

**DOI:** 10.1097/MD.0000000000038569

**Published:** 2024-06-21

**Authors:** Elvan Ocmen, Ismail Erdemir, Hale Aksu Erdost, Volkan Hanci

**Affiliations:** a Dokuz Eylul University Medical Faculty, Department of Anesthesiology and Reanimation, Balcova, Izmir, Turkey; b Sincan Training Hospital Department of Anesthesiology and Reanimation, Balcova, Izmir, Turkey.

**Keywords:** children, health information, internet, pain, pediatric, readability

## Abstract

We aimed to examine the patient education materials (PEMs) on the internet about “Child Pain” in terms of readability, reliability, quality and content. For our observational study, a search was made on February 28, 2024, using the keywords “Child Pain,” “Pediatric Pain,” and “Children Pain” in the Google search engine. The readability of PEMs was assessed using computer-based readability formulas (Flesch Reading Ease Score [FRES], Flesch-Kincaid Grade Level [FKGL], Automated readability index (ARI), Gunning Fog [GFOG], Coleman-Liau score [CL], Linsear Write [LW], Simple Measure of Gobbledygook [SMOG]). The reliability and quality of websites were determined using the Journal of American Medical Association (JAMA) score, Global Quality Score (GQS), and DISCERN score. 96 PEM websites included in our study. We determined that the FRES was 64 (32–84), the FKGL was 8.24 (4.01–15.19), ARI was 8.95 (4.67–17.38), GFOG was 11 (7.1–19.2), CL was 10.1 (6.95–15.64), LW was 8.08 (3.94–19.0) and SMOG was 8.1 (4.98–13.93). The scores of readability formulas showed that, the readability level of PEMs was statistically higher than sixth-grade level with all formulas (*P* = .011 for FRES, *P* < .001 for GFOG, *P* < .001 for ARI, *P* < .001 for FKGL, *P* < .001 for CL and *P* < .001 for SMOG), except LW formula (*P* = .112). The websites had moderate-to-low reliability and quality. Health-related websites had the highest quality with JAMA score. We found a weak negative correlation between Blexb score and JAMA score (*P* = .013). Compared to the sixth-grade level recommended by the American Medical Association and the National Institutes of Health, the readability grade level of child pain-related internet-based PEMs is quite high. On the other hand, the reliability and quality of PEMs were determined as moderate-to-low. The low readability and quality of PEMs could cause an anxious parent and unnecessary hospital admissions. PEMs on issues threatening public health should be prepared with attention to the recommendations on readability.

**What known**

Internet is widely used for health information by individuals. However, internet-based patient education materials are usually difficult to understand by general population.

**What new**

We evaluated the readability and reliability of websites related to child pain. We found that the readability of websites is below recommended level of sixth-grade level. Most websites had a reliability and quality level of moderate-to-low.

## 1. Introduction

Pain is a disturbing problem for both children and their parents. Pain can impair quality of life of both the individual and family. Untreated pain can result with chronic pain and chronic pain prevalence has been reported between 11% and 53% in children.^[1]^ Pediatric pain can result with anxiety, depression, low self-esteem, the child can miss school and social or sportive activities. It can also create great concern and desperation among families because they may not know the cause of the pain or how to cure it.^[1]^

Almost every individual has a smartphone or computer so internet is generally the first option for people to take health information.^[2]^ This fact increases the number of health-related websites. It has been reported that there were more than 70000 health-related websites.^[3,4]^ Internet is an important and valuable source for parents for searching health information. The 2018 Health Information National Trends Survey reported that internet is the first choice of health information resource for 74.5% of their respondents.^[5,6]^ But the quality and readability of internet health information resources varies. Patient education materials (PEMs) should be readable and understandable for public. National Institutes of Health, American Medical Association and The US Department of Health and Human Services recommend that internet-based PEMs should be below sixth-grade level.^[7]^ But unfortunately, several studies that investigate the readability of health information resources showed that many of them are higher than recommended level.^[8]^

Parents seek for online information which is easy to access, up to date and generally from a hospital website. It has been shown that 1 of 8 parents first search internet before going to a hospital emergency room.^[8,9]^ This finding shows that, it is important to conduct readable and understandable online health information. Especially for parents that are concerned with their child health, it is more important to understand the PEMs to lower the anxiety, unnecessary hospital admissions or the risk of underestimating serious conditions. However, we did not find any study assessing the internet-based PEMs on Child Pain, which is a very important issue for public health. We hypothesized that the readability, quality and reliability of internet-based PEMs related to pain in children were higher than the recommended levels. In order to test this hypothesis, we evaluated the levels of these parameters of internet-based PEMs related to Child Pain.

## 2. Methods

We designed this cross-sectional, observational study to evaluate the readability and quality of internet-based PEMs about child pain. After approval of Dokuz Eylul University Hospital noninvasive Studies Ethics Committee (2024/08–10), on February 28, 2024, the terms “Child Pain” “Pediatric Pain” and “Pain in Children” were searched on Google (https://www.google.com) search engine. Google search engine was preferred and used because it is the most preferred search engine.^[10]^

In order to avoid search results being affected, before our search in Google search machine, web browsing history was erased and cookies were deleted. At the same time, we signed out from all Google accounts. Google Incognito mode was used. The uniform resource locators (URLs) of the websites were recorded.^[11–14]^

The websites that are not related to pain in children, has advertisement marking, need subscription or registration, are not in English, the scientific papers which do not include patient information, the sale sites that do not have a patient information section, the ones include video or audio content without a text and duplicate websites were not included in our study. Video contents, figures and tables, pictures, punctuation marks, website URLs, references, telephone, address and author information in the text were also excluded while evaluating the texts in order to prevent false results.^[11,12,15]^

If no evaluation criteria were on home page of the website, the 3-click rule was used. The 3-click rule is; the user should reach information with up to 3 mouse clicks, it is thought that unless they reach information in 3 clicks the users leave that website.^[11,12,15]^

The readability and quality were evaluated by 2 independent researchers (V.H., I.E.) with proper measurement tools. The data was evaluated by 2 different researchers independently, after training how to use the measurement tools, to avoid bias.

### 2.1. Website typology

The websites were classified according to their ownership and types.^[11,12,16]^ The URL extensions (.com,.org,.edu,.gov and.net) were recorded and assessed.^[17]^ Typologies of the websites were classified as society/professional, health-related, government, hospital, news websites and others.^[11,12,18]^

### 2.2. Website rank values

The websites’ rank values were recorded and evaluated for each website using the extensions of the “google” website, “WebRankStats” (http://www.webrankstats.com), and “Blexb” (https://www.blexb.com).^[11,12]^

### 2.3. Reliability and quality of websites

The Journal of American Medical Association (JAMA) Benchmark criteria was used to evaluate websites.^[11–13,19]^ It assesses 4 items; authorship, attribution, disclosure and currency. The presence of each item adds 1 point to the score. JAMA Quality test criteria, Modified DISCERN and Global Quality Score (GQS) were used to assess the quality of the websites as previous studies.^[11,12,20]^ Modified DISCERN includes 5 questions addressing the quality of the website and each “yes” answer is 1 point where higher scores indicate higher reliability and quality. GQS is a 5-point scale where 1 point is poor quality and 5 points is excellent quality.

### 2.4. Readability

The texts of the websites were transferred to Microsoft Office Word 2007 (Microsoft Corporation, Redmond, WA). In order to analyze the readability, as in previous studies about readability, the punctuation marks, excluding periods, were erased.^[11,12,17]^ The texts were evaluated by using “www.readability-score.com” and the formulas; The Flesch Reading Ease Score (FRES), Flesch-Kincaid grade level (FKGL), Simple Measure of Gobbledygook (SMOG), Gunning Fog (GFOG), Coleman-Liau score (CL), automated readability index (ARI) and Linsear Write (LW) were used to assess readability.^[11,12,17]^ All formulas were used for each PEM in our study. These are the most popular formulas used while assessing the readability of texts and the ones commonly used in similar articles.^[11,12,17]^ FRES formula uses the ratio of total number of words to total number of sentences and the ratio of total number of syllables to total number of words. FKGL analyzes the sentence length and word complexity. Longer sentences and words with more syllables indicate a higher difficulty level. The SMOG Index focuses on polysyllabic words, which often indicate complex vocabulary and sentence structures. The GFOG formula calculates a grade level based on the average sentence length and the percentage of complex words. CL focuses on the average number of letters and sentences, assuming that longer words and longer sentences are more difficult to read. The ARI formula assesses the average number of characters per word and the average number of words per sentence. LW formula was created by The US Air Force to evaluate the readability of their technical manuals. It calculates the grade level of the text by evaluating the sentence length and the number of words with 3 or more syllables. The readability scores were compared to sixth-grade readability level.^[11,12]^ This readability level is ≥ 60.0 for FRES formula and < 7 for other used formulas.^[11,12]^

### 2.5. Content analysis

Contents of PEMs about child pain were evaluated. In the contents; definition, pathophysiology, incidence, epidemiology, risk factors, diagnosis, treatment, pain scores, morbidity, mortality, complications and prevention were evaluated.^[11,12]^

### 2.6. Statistical analysis

SPSS Windows 25.0 (SPSS Inc., Chicago, IL) was used for statistical analysis. Frequency data were given as numbers (n) and percentages (%). Data taking continuous values were represented as median (minimum-maximum). Frequency variables were compared with Chi-square or Fisher exact tests. The data taking continuous values were assessed with Kruskal-Wallis or Mann-Whitney U tests. Correlation analysis was made with Spearman correlation coefficient. *P* value of <.05 was determined as significant. “Bonferroni adjustment” was used in multi-group analyses, and the *P* value was determined by the number of groups.

## 3. Results

The search using Google showed 1.5 million results. The records of the first 167 websites were included in our study. After the first 167 websites, the duplications started. 20 websites (12%) including scientific materials, 6 (3.6%) unrelated websites, 4 (2.4%) commercial websites, 4 (2.4%) websites without texts and 36 (21.6%) websites that could not be reached were excluded from the study. 96 websites that met the inclusion criteria were analyzed.

### 3.1. Website typologies

In 96 websites included in our study, hospital websites (n = 44, 45.8%) have the highest percentage, the second most common typology was health-related websites (n = 18, 18.8%) (Table 1).

**Table 1:** Comparison of Blexb, JAMA, DISCERN, GQS scores, presences and reading levels according to the typologies of the websites.

	Society/Professional	Hospital	Health related	News	Government	Other	*P*
n(%)	13 (13.5%)	44 (45.8%)	18 (18.8%)	3 (3.1%)	14 (14.6%)	4 (4.2%)	
FRES	64 (32–84)	62.5 (33–81)	60 (33–76)	59 (49–60)	71.5 (59–80)	57 (36–66)	.019
GFOG	11.8 (7.1–15.7)	11.05 (7.7–19.2)	10.85 (8.1–17.6)	12.4 (10.3–15.5)	9 (7.3–11.9)	12.4 (10–14.7)	.02
FKGL	8.5 (4.01–12.72)	8.26 (5.01–15.19)	8.51 (5.35–13.98)	9.65 (7.61–12.16)	6.35 (5.1–8.73)	9.45 (7.6–11.48)	.02
CL	10.31 (6.95–15.64)	10.32 (7.73–14.15)	10.18 (7.47–14.22)	10.39 (10.21–12.34)	8.53 (7.22–11.93)	11.09 (9.45–14.81)	.015
SMOG	8.75 (5.34–11.25)	8.13 (5.74–13.93)	8.06 (6.17–12.79)	8.99 (7.55–11.24)	6.81 (4.98–8.79)	8.99 (7.44–10.76)	.03
ARI	9.49 (4.67–13.86)	9.28 (5.42–17.38)	9.24 (5.81–16.39)	11.04 (7.68–14.47)	6.98 (5.06–9.94)	10.43 (8.24–12.60)	**.008**
LW	10.46 (4.63–13.71)	8.14 (3.94–19)	9.22 (4.33–18.13)	11.83 (5.9–15.07)	6.37 (4.37–10.63)	8.41 (7.74–12.36)	.081
Blexb Median (min-max)	1740954 (45825–9388825)	261960.5 (903–27939958)	251951.5 (256–11585701)	100 (98–1066)	71253 (819–5083320)	1604 (11–234736)	**<.001**
JAMA Median (min-max)	1 (0–4)	1 (0–3)	2 (1–4)	2 (2–2)	1 (0–2)	1 (1–2)	**.001** .021
Insufficient data (0/1 point)	8 (61.8%)	34 (77.3%)	6 (33.3%)	0 (0%)	11 (78.6%)	3 (75%)	
Partially sufficient data (2/3 p)	4 (30.8%)	10 (22.7%)	11 (61.1%)	3 (100%)	3 (21.4%)	1 (25%)	
Completely sufficient data (4 point)	1 (7.7%)	0 (0%)	1 (5.6%)	0 (0%)	0 (0%)	0 (0%)	
DISCERN Median (min-max)	2 (0–5)	2 (0–4)	3 (1–5)	2 (1–4)	2 (1–4)	2.5 (1–4)	.065 .397
0 point (%)	2 (15.4%)	2 (4.5%)	0 (0%)	0 (0%)	0 (0%)	0 (0%)	
1 point (%)	1 (7.7%)	14 (31.8%)	1 (5.6%)	1 (33.3%)	2 (14.3%)	1 (25%)	
2 point (%)	5 (38.5%)	18 (40.9%)	7 (38.9%)	1 (33.3%)	8 (57.1%)	1 (25%)	
3 point (%)	2 (15.4%)	9 (20.5%)	6 (33.3%)	0 (0%)	3 (21.4%)	1 (25%)	
4 point (%)	2 (15.4%)	1 (2.3%)	3 (16.7%)	1 (33.3%)	1 (7.1%)	1 (25%)	
5 point (%)	1 (7.7%)	0 (0%)	1 (5.6%)	0 (0%)	0 (0%)	0 (0%)	
GQS Median (min-max)	2 (1–5)	2 (1–5)	3 (2–5)	2 (1–4)	3 (1–4)	3 (1–4)	.371 .302
Low quality (1/2 point)	7 (53.9%)	25 (56.8%)	6 (33.3%)	2 (66.6%)	5 (35.7%)	2 (50%)	
Medium quality (3 point)	4 (30.8%)	12 (27.3%)	5 (27.8%)	0 (0%)	7 (50%)	0 (0%)	
High quality (4/5 point)	2 (15.4%)	7 (15.9%)	7 (39.1%)	1 (33.3%)	2 (14.3%)	2 (50%)	
Reading level							.028
Fairly easy to read n(%)	1 (7.7%)	3 (6.8%)	0 (0%)	0 (0%)	4 (28.6%)	0 (0%)	
Standard/Average n(%)	2 (15.4%)	11 (25%)	6 (33.3%)	0 (0%)	6 (42.9%)	0 (0%)	
Fairly difficult to read n(%)	7 (53.8%)	25 (56.8%)	7 (38.9%)	2 (66.7%)	3 (21.4%)	2 (50%)	
Difficult to read n(%)	2 (15.4%)	3 (6.8%)	1 (5.6%)	0 (0%)	0 (0%)	2 (50%)	
Very difficult to read n(%)	1 (7.7%)	2 (4.5%)	2 (11.1%)	1 (33.3%)	1 (7.1%)	0 (0%)	
Impossible to comprehend	0 (0%)	0 (0%)	2 (11.1%)	0 (0%)	0 (0%)	0 (0%)	
Readers age							.103
8–9 years old (fourth and fifth graders) n(%)	1 (7.7%)	0 (0%)	0 (0%)	0 (0%)	0 (0%)	0 (0%)	
10–11 years old (fifth and sixth graders) n(%)	0 (0%)	2 (4.5%)	0 (0%)	0 (0%)	3 (21.4%)	0 (0%)	
11–13 years old (sixth and seventh graders) n(%)	1 (7.7)	7 (15.9 %)	2 (11.1%)	0 (0%)	5 (35.7%)	0 (0%)	
12–14 years old (seventh and eighth graders) n(%)	1 (7.7%)	3 (6.8%)	3 (16.7%)	0 (0%)	2 (14.3%)	0 (0%)	
13–15 years old (eighth and ninth graders) n(%)	1 (7.7%)	9 (20.5%)	3 (16.7%)	0 (0%)	1 (7.1%)	0 (0%)	
14–15 years old (ninth to tenth graders) n(%)	4 (30.8%)	11 (25%)	1 (5.6%)	1 (33.3%)	3 (21.4%)	2 (50%)	
15–17 years old (tenth to eleventh graders) n(%)	2 (15.4%)	7 (15.9%)	4 (22.2%)	1 (33.3%)	0 (0%)	0 (0%)	
17–18 years old (twelfth graders) n(%)	2 (15.4%)	3 (6.8%)	1 (5.6%)	0 (0%)	0 (0%)	2 (50%)	
18–19 years old (college level entry) n(%)	1 (7.7%)	1 (2.3%)	2 (11.1%)	1 (33.3%)	0 (0%)	0 (0%)	
21–22 years old (college level)	0 (0%)	0 (0%)	2 (11.1%)	0 (0%)	0 (0%)	0 (0%)	
College graduate n(%)	0 (0%)	1 (2.3%)	0 (0%)	0 (0%)	0 (0%)	0 (0%)	

Bold character; statistically different (*P* < .0083, Bonferroni adjustment).

ARI = automated readability index, CL = Coleman-Liau score, FKGL = Flesch-Kincaid grade level, FRES = Flesch reading ease score, GFOG = Gunning FOG, GQS = Global Quality Score, JAMA = Journal of American Medical Association Benchmark Criteria, LW = Linsear Write, SMOG = Simple Measure of Gobbledygook.

### 3.2. Readability evaluation

The calculated values of readability scores of evaluated 96 websites were; FRES 64 (32–84), GFOG 11 (7.1–19.2), ARI 8.95 (4.67–17.38), FKGL 8.24 (4.01–15.19), CL 10.1 (6.95–15.64), SMOG 8.10 (4.98–13.93) and LW 8.08 (3.94–19.0) (Table 2). The readability of all 96 websites was assessed by all 7 formulas. The readability of the websites was significantly higher than sixth-grade reading level with all formulas (*P* = .011 for FRES, *P* < .001 for GFOG, *P* < .001 for ARI, *P* < .001 for FKGL, *P* < .001 for CL and *P* < .001 for SMOG) except the LW formula (*P* = .112) (Table 2).

**Table 2:** Top 10 and other than top 10 websites’ mean reading indices and statistical comparison of text content to 6th-grade reading level, Blexb rank, JAMA, DISCERN, GQS, and typology results.

	Top 10 (n = 10)	Others (n = 86)	Total (n = 96)	*P* *	*P* **
Readability indexes	Median (min-max)	Median (min-max)	Median (min-max)		
FRES	65.5 (39–78)	63.5 (32–84)	64.0 (32–84)	.606	**.011**
GFOG	10.35 (8–13.9)	11.25 (7.1–19.2)	11.0 (7.1–19.2)	.475	**<.001**
FKGL	7.68 (5.27–11.37)	8.28 (4.01–15.19)	8.24 (4.01–15.19)	.598	**<.001**
CL	10.21 (7.22–14.38)	10.08 (6.95–15.64)	10.10 (6.95–15.64)	.905	**<.001**
SMOG	7.77 (6.07–10.07)	8.24 (4.98–13.93)	8.10 (4.98–13.93)	.513	**<.001**
ARI	8.48 (5.24–12.31)	9.15 (4.67–17.38)	8.95 (4.67–17.38)	.769	**<.001**
LW	7.79 (5.71–12.69)	8.25 (3.94–19.0)	8.08 (3.94–19.0)	.589	.112
Grade level	9.0 (6.0–12.0)	9.0 (1.0–16.0)	9.0 (1.0–16.0)	.794	**<.001**
Blexb	77134.0 (756.0–5834129.0)	172689.5 (11.0–27939958)	158745.0 (11.0–27939958.0)	.154	---
JAMA [Median(Min-Max)]	1 (0–3)	1 (0–4)	1 (0–4)	.435	--
DISCERN [Median(Min-Max)]	2.5 (0–4)	2 (0–5)	2 (0–5)	.282	--
GQS [Median(Min-Max)]	4 (1–5)	2 (1–5)	3 (1–5)	**.002**	--
JAMA	**n(%**)	**n(%**)	**n(%**)	.812	--
Insufficient data (0/1 point)	6 (60%)	56 (65.1%)	62 (64.5%)		
Partially sufficient data (2/3 p)	4 (40%)	28 (32.5%)	32 (33.3%)		
Completely sufficient data (4 point)	0 (0%)	2 (2.3%)	2 (2.1%)		
DISCERN	**n(%**)	**n(%**)	**n(%**)	.397	---
0 point (%)	1 (10%)	3 (3.5%)	4 (4.2%)		
1 point (%)	0 (0%)	20 (23.3%)	20 (20.8%)		
2 point (%)	4 (40%)	36 (41.9%)	40 (41.7%)		
3 point (%)	4 (40%)	17 (19.8%)	21 (21.9%)		
4 point (%)	1 (10%)	8 (9.3%)	9 (9.4%)		
5 point (%)	0 (0%)	2 (2.3%)	2 (2.1%)		
GQS	**n(%**)	**n(%**)	**n(%**)	**<.001**	---
Low quality (1/2 point)	1 (10%)	46 (53.5%)	47 (49%)		
Medium quality (3 point)	2 (20%)	26 (30.2%)	28 (29.2%)		
High quality (4/5 point)	7 (70%)	14 (16.3%)	21 (21.9%)		
Typology	**n(%**)	**n(%**)	**n(%**)	.891	---
Society/Professional	1 (10%)	12 (14%)	13 (13.5%)		
Hospital	4 (40%)	40 (46.5%)	44 (45.8%)		
Health related	2 (20%)	16 (18.6%)	18 (18.8%)		
News	0 (0%)	3 (3.5%)	3 (3.1%)		
Government	2 (20%)	12 (14%)	14 (14.6%)		
Other	1 (10%)	3 (3.5%)	4 (4.2%)		

Bold character; statistically different (*P* < .05).

ARI = automated readability index, CL = Coleman-Liau score, FKGL = Flesch-Kincaid grade level, FRES = Flesch reading ease score, GFOG = Gunning FOG, GQS = Global Quality Score, JAMA = Journal of American Medical Association Benchmark Criteria, LW = Linsear Write, SMOG = Simple Measure of Gobbledygook

* Comparison of the first 10 websites and remaining 86 websites according to parameters.

** Comparison of the 96 websites’ according to 6th grade reading level.

**Figure 1.**
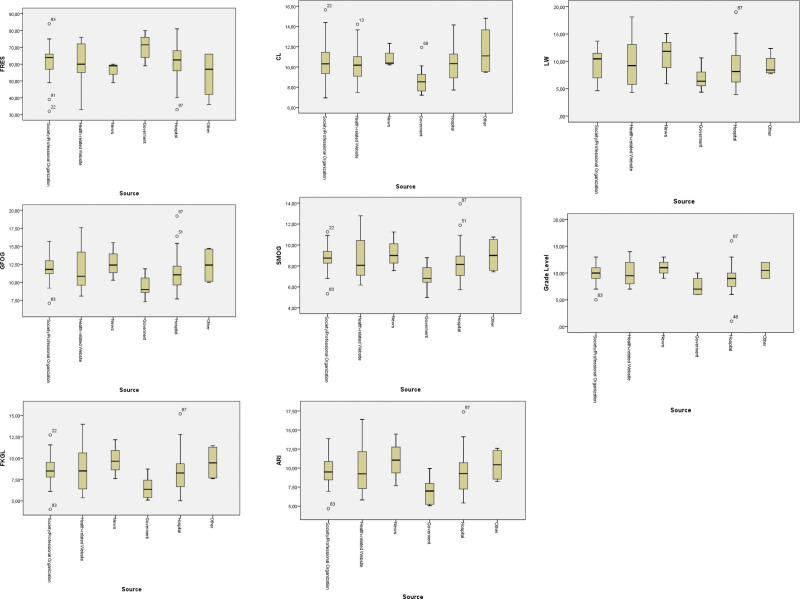
Readability scores according to website typologies.

We found a statistically significant difference between websites’ typologies with only ARI formula (*P* = .008) (Table 1) (Fig. 1).

### 3.3. Reliability and quality evaluation

**Figure 2.**
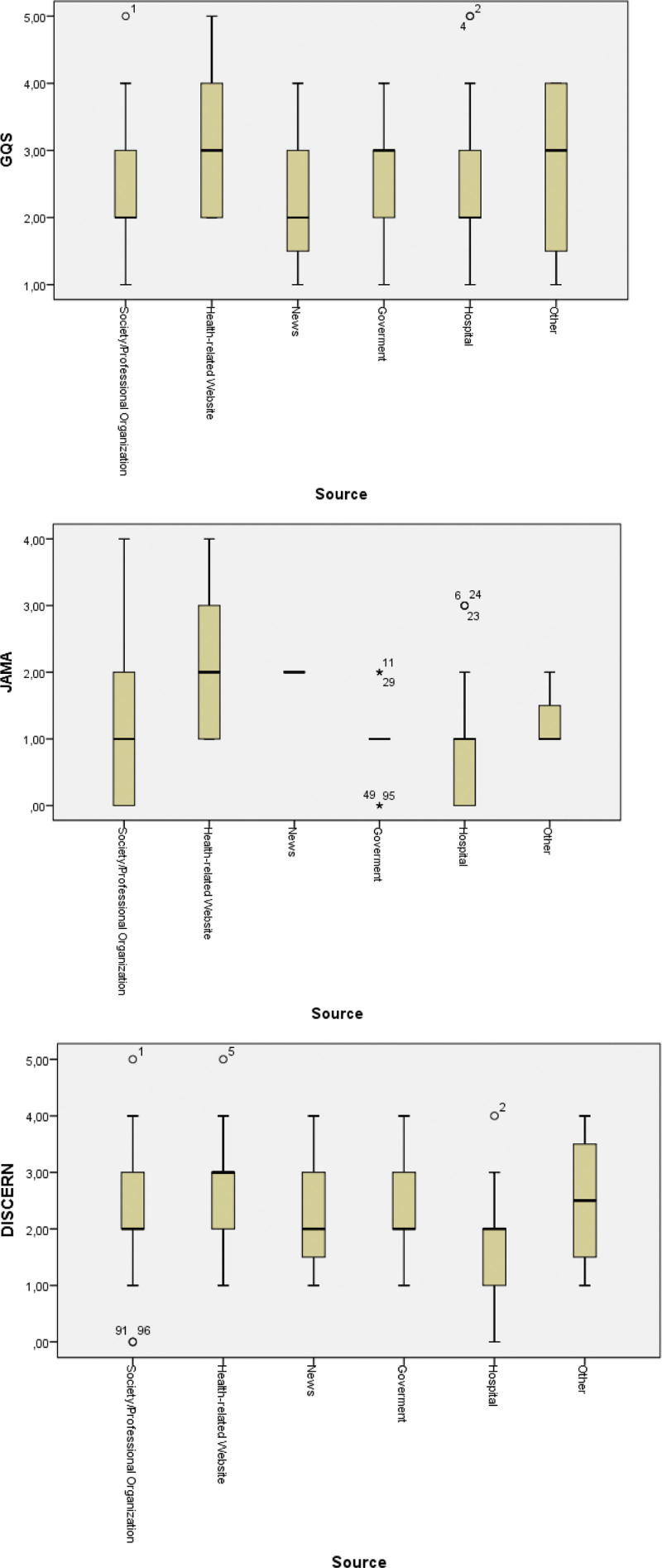
GQS, JAMA and DISCERN scores according to website typologies. GQS: Global Quality Score, JAMA: Journal of American Medical Association.

The JAMA score for 96 websites was 1 (0–4), GQS score was 3 (1–5) and DISCERN score was 2 (0–5). 75 (78.2%) of 96 websites’ GQS score was ≤ 3, JAMA score of 86 (89.5%) websites was ≤ 2 and DISCERN score of 64 (66.7%) websites was ≤ 2 indicating that they have a moderate-to-low reliability and quality. We could not find any difference between typology of the websites with GQS and DISCERN scores. But there was a statistically significant difference with JAMA score (*P* = .009) (Table 2) (Fig. 2). The quality of websites with JAMA score can be aligned as; health-related > society/professional organization > hospital > news > other > government websites.

### 3.4. Correlation analysis

We found a weak negative correlation between JAMA score and Blexb score (*P* = .013) but we could not find any correlation with GQS and DISCERN scores (*P* > .05). A weak correlation was found between LW score and Blexb score (*P* = .043). No correlation was observed between Blexb and other readability scores (*P* > .05) (Table 3).

**Table 3:** Correlation relationships between rank and readability formulas, Blexb, JAMA, DISCERN, GQS scores.

	Blexb		GQS		JAMA		DISCERN	
	r	*P*	r	*P*	r	*P*	r	*P*
Blexb	----	----	−0.186	.070	−0.253	**.013**	−0.188	.066
GQS	−0.186	.070	----	----	0.335	**.001**	0.778	**<.001**
JAMA	−0.253	**.013**	0.335	**.001**	----	----	0.520	**<.001**
DISCERN	−0.188	.066	0.778	**<.001**	0.520	**<.001**	----	----
FRES	0.025	.807	0.027	.793	−0.299	**.003**	−0.121	.239
GFOG	0.146	.157	−0.042	.686	0.270	**.008**	0.082	.429
FKGL	0.118	.252	−0.047	.650	0.332	**.001**	0.088	.392
CL	−0.059	.569	−0.042	.684	0.254	**.013**	0.135	.190
SMOG	0.129	.211	−0.045	.661	0.264	**.009**	0.085	.411
ARI	0.128	.214	−0.059	.567	0.330	**.001**	0.079	.447
LW	0.207	**.043**	−0.048	.641	0.281	**.006**	0.044	.673
Grade Level	0.089	.389	−0.065	.531	0.350	**<.001**	0.128	.214

Bold character; statistically different (*P* < .05).

Spearman's correlation analyses.

r: Spearman's correlation coefficient.

ARI = automated readability index, CL = Coleman-Liau score, FKGL = Flesch-Kincaid grade level, FRES = Flesch reading ease score, GFOG = Gunning FOG, JAMA = Journal of American Medical Association Benchmark Criteria, LW = Linsear Write, SMOG = Simple Measure of Gobbledygook.

### 3.5. Content analysis

Each topic related to child pain was assessed in content analysis and their frequencies were; definition 38.5%, pathophysiology 10.4%, incidence 11.5%, epidemiology 22.9%, risk factors 26.0%, diagnosis 68.8%, treatment 72.9%, pain scores 22.9%, complications 24.0%, morbidity 9.4%, prevention 19.8%. Mortality was not mentioned in any of the websites. We did not observe a significant relation between website typologies and contents (*P* > .05) Table 4).

**Table 4:** Content analysis by typology.

		Society/Professional	Hospital	Health related	News	Government	Other	*P*
Definition	No	9 (69.2%)	30 (68.2%)	9 (50%)	2 (66.7%)	7 (50%)	2 (50%)	.669
	Yes	4 (30.8%)	14 (31.8%)	9 (50%)	1 (33.3%)	7 (50%)	2 (50%)	
Pathophysiology	No	11 (84.6%)	43 (97.7%)	14 (77.8%)	3 (100%)	12 (85.7%)	3 (75%)	.177
	Yes	2 (15.4%)	1 (2.3%)	4 (22.2%)	0 (0%)	2 (14.3%)	1 (25%)	
Incidence	No	12 (92.3%)	41 (93.2%)	12 (66.7%)	3 (100%)	13 (92.9%)	4 (100%)	.056
	Yes	1 (7.7%)	3 (6.8%)	6 (54.5%)	0 (0%)	1 (7.1%)	0 (0%)	
Epidemiology	No	10 (76.9%)	40 (90.9%)	11 (61.1%)	2 (66.7%)	9 (64.3%)	2 (50%)	.062
	Yes	3 (23.1%)	4 (9.1%)	7 (38.9%)	1 (33.3%)	5 (35.7%)	2 (50%)	
Risk factors	No	11 (84.6%)	37 (84.1%)	10 (55.6%)	2 (66.7%)	8 (57.1%)	3 (75%)	.135
	Yes	2 (15.4%)	7 (15.9%)	8 (44.4%)	1 (33.3%)	6 (42.9%)	1 (25%)	
Diagnosis	No	3 (23.1%)	13 (29.5%)	6 (33.3%)	1 (33.3%)	6 (42.9%)	1 (25%)	.918
	Yes	10 (76.9%)	31 (70.5%)	12 (66.7%)	2 (66.7%)	8 (57.1%)	3 (75%)	
Treatment	No	4 (30.8%)	12 (27.3%)	6 (33.3%)	2 (66.7%)	0 (0%)	2 (50%)	.106
	Yes	9 (69.2%)	32 (72.7%)	12 (66.7%)	1 (33.3%)	14 (100%)	2 (50%)	
Pain scores	No	8 (61.5%)	32 (72.7%)	17 (94.4%)	3 (100%)	12 (85.7%)	2 (50%)	.132
	Yes	5 (38.5%)	12 (27.3%)	1 (5.6%)	0 (0%)	2 (14.3%)	2 (50%)	
Complications	No	12 (92.3%)	34 (77.3%)	13 (72.2%)	2 (66.7%)	10 (71.4%)	2 (50%)	.569
	Yes	1 (7.7%)	10 (22.7%)	5 (27.8%)	1 (33.3%)	4 (28.6%)	2 (50%)	
Morbidity	No	13 (100%)	41 (93.2%)	15 (83.3%)	3 (100%)	13 (92.9%)	2 (50%)	.052
	Yes	0 (0%)	3 (6.8%)	3 (16.7%)	0 (0%)	1 (7.1%)	2 (50%)	
Prevention	No	11 (84.6%)	37 (84.1%)	14 (77.8%)	3 (100%)	10 (71.4%)	2 (50%)	.498
	Yes	2 (15.4%)	7 (15.9%)	4 (22.2%)	0 (0%)	4 (28.6%)	2 (50%)	

### 3.6. Comparison of the first 10 and other websites

The first 10 websites’ Blexb rank values were not different from the rank values of other 86 websites (*P* = .891).

**Figure 3.**
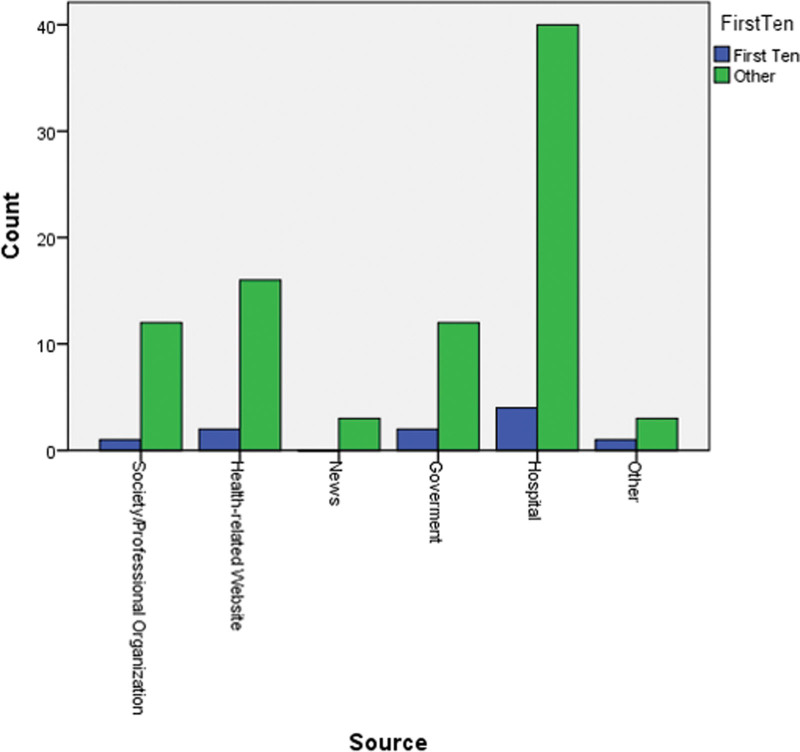
Website typologies distribution of top 10 and other websites.

Also, we could not find any difference between the typologies of the first 10 and the other 86 websites (Fig. 3).

When the quality of first 10 websites was compared with other websites we found a statistically significant difference in GQS score (*P* = .02). Similarly, there was a significant difference in GQS quality score distribution between the first 10 websites and others (*P* = .02). The JAMA and DISCERN scores of first 10 websites were similar to other 86 websites (*P* = .435 and *P* = .282, respectively). The distribution percentages of JAMA and DISCERN scores of first 10 websites and others were not different (*P* = .761 and *P* = .397, respectively).

Reading levels of first 10 websites were not different than other websites (*P* = .645). When readability was assessed between first 10 and other websites there was not any difference with ARI (*P* = .769), FRES (*P* = .606), GFOG (*P* = .475), FKGL (*P* = .598), CL (*P* = .905), SMOG (*P* = .513), or LW (*P* = .589) scores (Table 2).

Treatment (n = 8), diagnosis (n = 7), and complications (n = 5) were most common contents in the first 10 websites. We could not find any difference between the contents of first 10 websites and the others (*P* > .05) (Table 5).

**Table 5:** Content analysis of websites about child pain.

Content		First 10 websites	Other 86 websites	Total	*P*
Definition	No	6 (60%)	53 (61.6%)	59 (61.5%)	.587
	Yes	4 (40%)	33 (38.4%)	37 (38.5%)	
Pathophysiology	No	10 (100%)	76 (88.4%)	86 (89.6%)	.314
	Yes	0 (0%)	10 (11.6%)	10 (10.4%)	
Incidence	No	8 (80%)	77 (89.5%)	85 (88.5%)	.321
	Yes	2 (20%)	9 (10.5%)	11 (11.5%)	
Epidemiology	No	7 (70%)	67 (77.9%)	74 (77.1%)	.412
	Yes	3 (30%)	19 (22.1%)	22 (22.9%)	
Risk factors	No	8 (80%)	63 (73.3%)	71 (74%)	.489
	Yes	2 (20%)	23 (26.7%)	25 (26%)	
Diagnose	No	3 (30%)	27 (31.4%)	30 (31.3%)	.619
	Yes	7 (70%)	59 (68.6%)	66 (68.8%)	
Treatment	No	2 (20%)	24 (27.9%)	26 (27.1%)	.457
	Yes	8 (80%)	62 (72.1%)	70 (72.9%)	
Pain Scores	No	7 (70%)	67 (77.9%)	74 (77.1%)	.412
	Yes	3 (30%)	19 (22.1%)	22 (22.9%)	
Complications	No	5 (50%)	68 (79.1%)	73 (76%)	.056
	Yes	5 (50%)	18 (20.9%)	23 (24%)	
Morbidity	No	7 (70%)	80 (93.0%)	87 (90.6%)	.050
	Yes	3 (30%)	6 (7.0%)	9 (9.4%)	
Prevention	No	7 (70%)	70 (81.4%)	77 (80.2%)	.312
	Yes	3 (30%)	16 (18.6%)	19 (19.8%)	

## 4. Discussion

We found that the readability grade level of internet-based PEMs about “Child Pain” was significantly higher than the recommended sixth-grade level. We also showed that most of the PEMs’ reliability and quality levels were low to moderate. Hospitals’ websites were the most frequent websites and there was not a difference between the first 10 and other websites typologies and readability.

Pain is an unpleasant feeling that can be associated with tissue damage.^[21]^ It can be in an acute or chronic nature. In Germany, the prevalence of pain has been reported as 64.5% for children between 3 and 10 years and 77.6% for 11 to 17 years.^[22]^ The incidence of chronic or recurrent pain has been reported between 11% and 53%.^[1]^ These results show us that more than half of the children suffer from acute or chronic pain. In a recent review, the authors summarized the epidemiologic studies and found that one-third of the children had stomach aches and 20% of the children had inadequate pain relief therapy after surgery.^[21]^ It has been shown that pain in pediatric age can result with impairment of cognitive and social development.^[23,24]^ Pain experience can be stressful and even traumatizing for the child and parents. Anxiety, depression and low self-esteem can arise as a result of pediatric pain. Pain is also the reason of concern and desperation of the child and parents.^[1]^

Nowadays internet is the primary source of health information for many individuals.^[25]^ It was shown that 90% of the people who live in America use internet and health-related topics constitute 72% of internet searches.^[26]^ Nearly, 6.5 million Google searches are for accessing health information per day.^[27]^ For anxious parents searching internet for information about their child pain, the internet-based PEMs should be easy to read and understand. Otherwise, despite easy access to health information on internet, PEMs can cause confusion and anxiety.^[28]^

Especially for children suffering chronic pain, improving parent related factors is one of the key factors to improve child functioning.^[29,30]^ When the parents have increased levels of protective behaviors, children became more disable.^[31–34]^ Parent behaviors are usually related to their experiences and the information they have. In a recent meta-analysis, correlation between parent anxiety and child pain and disability has been shown.^[34]^ It is understandable that a misinformed parent would be more anxious and protective. This study showed that the PEMs about child pain are difficult to read and have moderate quality which could easily result with misinformed parents. Therefore, the easiest way for parents to access information, the internet-based PEMs, should be easy to read and understand.

Although the number of internet-based PEMs is increasing due to high demand, their readability and quality varies.^[4]^ Moreover, the understandability of PEMs depends on the person health literacy which is defined as to read and understand health information and use it while making health-related decisions. The adequate health literacy has been reported as 12% in American population.^[35]^ As long as the readers do not understand, the information and accuracy of the website are not important. When the patient in question is a child, the parents have to make a health-related decision on behalf of the child. This would increase the anxiety of the parents. As we determined in our study the PEMs about child pain are difficult to read so it is questionable that the PEMs could help an anxious parent to make health-related decisions. This is an important issue for children health and also for healthcare sector because a misinformed parent can decide to go to emergency for unnecessary pain or decide to stay home while urgent care is needed.

Readability is an objective measurement of written material understandability. There are several formulas to evaluate readability but the best one/ones to evaluate health-related materials are unknown.^[36]^ Therefore, in our study, we used 7 different formulas to evaluate the internet-based PEMs about “Child Pain” and also the estimated grade level. The recommended grade for readability is sixth-grade level. The United States Department of Health and Human Services considers below sixth-grade level as ‘easy to read’, 7 to 9^th^ grade level as “average difficulty” and above 9th grade level as “difficult.”^[37]^ This study showed that most of the internet-based PEMs about “Child Pain” are above sixth-grade level with all readability formulas except LW. Similar to our findings Arslan et al^[2]^ investigated the readability of PEMs about chest pain in children and found that their readability is higher than sixth-grade reading level. Our study, where we included the PEMs about general pain in children, indicates that the PEMs about “Child Pain” are far from understandable for families.

Another concern about the internet-based PEMs is their quality and reliability.^[9]^ Eysenbach et al^[38]^ reviewed the studies evaluating PEMs and they found that 70% of the studies reported a problem in the quality of the websites. In this study, similar to previous studies, we found that more than 2-third of the websites about “Child Pain” have low to moderate quality and reliability with JAMA, DISCERN and GQS scores.

Nearly half of the PEMs in our study were hospital websites unlike previous studies which found commercial websites have the greatest number.^[11–13,39]^ According to our findings the readability of the PEMs was significantly higher than sixth-grade level with ARI formula regardless of typology of the PEM. We also assessed the quality according to the typology of the website. Unlike other studies, we could not find a difference between websites according to their typology with DISCERN and GQS scores.^[11,12]^ But there was a significant difference with JAMA benchmark criteria which shows the health-related websites have the greatest quality whereas government websites have the lowest. Similar to our findings Arslan et al^[2]^ reported that physician and health information websites have highest JAMA scores. As mentioned before the readability of websites did not differ, so even though the health-related websites have high quality with JAMA scores we believe that they can not provide sufficient information for patients or families with low health literacy.

The statistics about Google reported that <10% of the people advance to the second page of the search results.^[40]^ Therefore we separately evaluated the first 10 results for readability and quality and compared the findings with other 86 results. The readability of first 10 websites was higher than 8-grade level and there was not a significant difference with other websites. Kocyigit et al^[16]^ and Bagcier et al^[41]^ could not find a difference between first 10 and other websites similar to our findings. Contrary to our findings, Basavakumar et al^[13]^ reported in their study that the first 10 websites were more readable than other websites. The GQS score showed that the quality of first 10 websites were higher than other websites. An interesting finding of this study was we could not find a difference at rank scores of first 10 and other websites. This finding may be result of low readability and quality of first 10 websites.

The most common contents of the PEMs about “Child Pain” were determined as “diagnosis” and “treatment.” This is an understandable finding since the most curious topics about pain are; the reason of the pain and how to treat it.

There are several limitations of this study. The first limitation is an unavoidable one and is related to internet nature. Internet is a dynamic information source so the ranks and search results may change. Second limitation is, we only search the websites in English. Including all languages would result in different results. Third and also an unavoidable limitation of our study is even though we cleaned the history and cookies of the search engine, the search results could still be related to our geographic location. In different regions of the world, the same keywords could result with different websites. Another limitation is evaluating readability with computer-based formulas. These formulas may overestimate the readability of the evaluated material. Although many studies assess the readability of PEMs with computer-based formulas, the ideal formula to evaluate readability of internet-based PEMs is unknown.^[2,4,5,7,8]^ We used 7 computer-based formulas to reduce the misleading results. The last limitation of our study is, we used only general search terms about child pain like “Child Pain,” “Pediatric Pain” and “Pain in Children.” We did not use the keywords for specific pain types like headache or postoperative pain because we wanted to include all kinds of pain that children may suffer and evaluate the PEMs about them. Our aim was to assess the readability and quality of PEMs about pain in childhood. Further studies could be planned to evaluate the PEMs about specific pain types in childhood.

In conclusion, we evaluated the readability and quality of internet-based PEMs and showed that the reliability and quality of websites are moderate-to-low and readability grade level is higher than the suggested sixth-grade level. We also found that the rank of first 10 websites did not differ from other websites and we believe that this could be as a result of low readability and quality of the websites. Our findings show that the PEMs that parents search for information about their child health are difficult to read and understand. We believe that while preparing PEMs, the readability and health literacy of general public should be considered by the editors of the websites. This would help parents to find satisfying information about their child health and decision-making. Further studies may be needed to explore the readability of PEMs about specific pain types that may affect a child well-being.

## Author contributions

**Conceptualization:** Elvan Ocmen, Volkan Hanci.

**Data curation:** Elvan Ocmen, Ismail Erdemir, Volkan Hanci.

**Formal analysis:** Volkan Hanci.

**Investigation:** Elvan Ocmen, Ismail Erdemir.

**Methodology:** Elvan Ocmen, Volkan Hanci.

**Writing – original draft:** Elvan Ocmen.

**Writing – review & editing:** Hale Aksu Erdost, Volkan Hanci.
